# An intracerebral microdialysis study to determine the neuropharmacokinetics of eribulin in patients with metastatic or primary brain tumors

**DOI:** 10.1007/s00280-024-04711-2

**Published:** 2024-10-18

**Authors:** Zeynep Eroglu, Timothy Synold, Behnam Badie, An Liu, Arnab Chowdhury, Julie Kilpatrick, Suzette Blanchard, Jana Portnow

**Affiliations:** 1 Present Address: City of Hope Comprehensive Cancer Center, Department of Medical Oncology and Therapeutics Research, Duarte, CA USA; 2 Department of Cancer Biology, Beckman Research Institute of City of Hope, Duarte, CA USA; 3 Department of Surgery, Division of Neurosurgery, City of Hope Comprehensive Cancer Center, Duarte, CA USA; 4 Department of Radiation Oncology, City of Hope Comprehensive Cancer Center, Duarte, CA USA; 5 Department of Computational and Quantitative Medicine, Beckman Research Institute of City of Hope, Duarte, CA USA; 6 Departmant of Clinical Research, City of Hope Comprehensive Cancer Center, Duarte, CA USA; 7 Present Address: Department of Cutaneous Oncology, Moffitt Cancer Center, Tampa, FL USA

**Keywords:** Eribulin, Intracerebral microdialysis, Brain metastases, Glioblastoma

## Abstract

**Purpose:**

Eribulin is an inhibitor of microtubule dynamics. It is not as highly protein bound as the taxanes and is less vulnerable to extrusion by P-glycoprotein in the blood–brain barrier (BBB). These features predict that eribulin could play an active role in managing brain tumors. Indeed, the small amount of published clinical data indicates eribulin may have some efficacy against breast cancer brain metastases. To better understand the potential of eribulin for treating brain tumors, we performed an intracerebral microdialysis study to determine the neuropharmacokinetics of eribulin in cancer patients undergoing tumor resection.

**Methods:**

After tumor removal, two microdialysis catheters were inserted into peritumoral brain tissue. Approximately 24 h after surgery, a single dose of eribulin 1.4 mg/m^2^ was administered intravenously. Dialysate samples were collected continuously for 72 h, with plasma samples collected in parallel. Eribulin concentrations were analyzed by tandem mass spectrometry.

**Results:**

Dialysate samples from 12 intracerebral microdialysis catheters placed in 7 study participants were included in the analysis. A statistically significant difference was observed between eribulin concentrations in brain tissue where BBB was disrupted versus intact, with a difference in mean maximum concentrations on log_2_ scale of 3.37 (std err = 0.59, *p*-value = 0.005). Nonetheless, overall brain to plasma ratios of eribulin only ranged from 0.13 to 1.99%.

**Conclusion:**

Although we could detect higher concentrations of eribulin in brain tissue where BBB was disrupted, intracerebral eribulin levels were not sufficient to predict eribulin would have consistent clinically meaningful activity against tumors in the brain.

**ClinicalTrials.gov Identifier:**

NCT02338037 (January 9, 2015).

**Supplementary Information:**

The online version contains supplementary material available at 10.1007/s00280-024-04711-2.

## Introduction

Despite advances in targeted agents and immunotherapies for the treatment of many solid tumors, survival rates remain low for most patients with either metastatic or malignant primary brain tumors. Radiation continues to be first-line therapy for many of these patients, as few chemotherapy agents can cross the blood–brain barrier to a significant degree.

Eribulin mesylate is approved by the Food and Drug Administration for the treatment of metastatic breast cancer and liposarcoma. It is a fully synthetic analog of halichondrin B, a natural product isolated from marine sponge. The main way in which eribulin produces cytotoxicity is by preventing microtubules from dividing; however, different from other microtubule inhibitors, such as taxanes, eribulin causes inhibition of the growth phase of microtubules but does not affect the shortening phase. Eribulin also inhibits mitotic spindle formation by causing development of non-productive tubulin, thereby decreasing the concentration of free tubulin in a cell [1]. In contrast to paclitaxel and docetaxel, which do not cross intact human blood–brain barrier, eribulin is not as highly protein-bound [2]. Moreover, in vitro data have shown that eribulin is less vulnerable to P-glycoprotein-mediated drug extrusion at the level of the blood–brain barrier than other microtubule inhibitors [3]. These differences indicate a potential role for eribulin in treating brain tumors.

Although preclinical distribution data in mice documented low penetration of eribulin into normal brain (i.e., through intact blood–brain barrier) [4], other preclinical in vivo studies demonstrated that eribulin can achieve cytotoxic concentrations in orthotopic brain tumor models where blood–brain barrier is locally disrupted due to growing tumor. Takahashi et al. [5], administered eribulin intraperitoneally to mice with intracerebral glioblastoma xenografts and found that eribulin suppressed tumor growth and prolonged survival. They also showed that intravenously administered eribulin penetrated the brain tumors and remained at a high concentration (91.7 nmol/L) for 24 h after injection, even when eribulin concentrations became undetectable in other organs and plasma. Additionally, we performed intracerebral microdialysis [6] in nude rats with orthotopic U87 human glioma who were treated with a single intravenous dose of eribulin 0.5 mg/kg, which is the human equivalent dose. We found that eribulin concentrations in tumor (average AUC 12.1 nmol/L x hr) were sufficient for cytotoxicity [7]. The measured average peak intracerebral concentration of eribulin (3.2 nmol) was also above the published IC_50_ of eribulin for a wide range of solid tumors, including breast cancer [8].

Published clinical experience with eribulin for treating brain tumors consists of multiple case reports [9–13], two small prospective observational studies [14, 15] and one small retrospective observational study [16], all describing the activity of eribulin against brain metastases from breast cancer. In the observational studies, intracranial objective response rates ranged from 5 to 16%. To further investigate the potential of eribulin for the treatment of brain tumors, we performed an intracerebral microdialysis study to define the neuropharmacokinetics of eribulin in patients with metastatic or primary brain tumors who were undergoing debulking craniotomies.

## Materials and methods

### Preparation for performing the intracerebral microdialysis study in brain tumor patients

An in vitro study was done to estimate the fractional recovery of eribulin by the microdialysis catheter. This in vitro assessment was performed with the same microdialysis equipment that would be used in the clinical trial. A microdialysis catheter with a molecular weight cutoff of 20 kDa (70 Brain MD Catheter; membrane length 10 mm, shaft length 100 mm; ref. no. P000050, M Dialysis) was placed into a reservoir containing a solution with a known concentration of eribulin. Artificial CSF (Perfusion Fluid CNS; ref. no. P000151, M Dialysis) perfused the microdialysis catheter at a flow rate of 1 µL/min while serial dialysate samples were collected.

The City of Hope Analytical Pharmacology Core Facility developed a quantitative assay for eribulin [17], and concentrations of eribulin in dialysate samples were determined by liquid chromatography/tandem mass spectrometry. The lower limit of detection of eribulin in brain extracellular fluid was set to 0.02 ng/ml. Recovery of eribulin by the microdialysis catheter at a flow rate of 1 µL/min was 100%, and so that was the flow rate used in the clinical trial.

### Study participants

Eligible patients were at least 18 years old, had either metastatic or primary brain tumor(s), and were planning to undergo resection of tumor. There was no limit to the number of prior treatments (any type of brain radiation, surgery, or chemotherapy) for their brain tumor(s). Adequate organ function was required and if corticosteroids were needed for controlling cerebral edema, patients had to be on a stable dose for at least 1 week prior to study enrollment. Participants also had to have a Karnofsky performance score ≥ 60.

### Study objectives

**Fig. 1:**
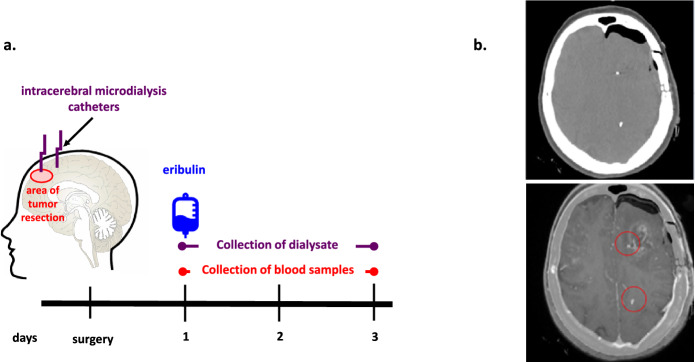
**a **Study Schema. During the surgery, after resection of tumor two microdialysis catheters were inserted in residual tumor and/or peritumoral brain tissue, as was technically feasible. Twenty four hours later a 1.4 mg/m^2^ dose of eribuin was admnistered to the patient intravenously. Serial dialysate and blood samples were collected during the following 48 h. **b** Representative post-operative imaging (from Participant 4**).** Top: gold tips of the microdialysis catheters are visible on a non-contrast CT scan. Bottom: merging of corresponding images from the participant’s post-operative non-contrast brain CT and MRI (T1 post-contrast sequence) scans show that one of the microdialysis catheter tips was placed in contrast-enhancing brain tissue (residual tumor) and the other one was placed in non-enhancing brain tissue (normal brain)

The primary objective of the study was to determine the neuropharmacokinetic profile of eribulin using intracerebral microdialysis. The secondary objective was to compare concentrations of eribulin in brain tissue where the blood–brain barrier was disrupted versus intact. This objective was accomplished by fusing the T1 post-contrast images from a participant’s post-operative brain MRI with their post-operative non-contrast CT scan of the brain in which the gold filament at the tip of the microdialysis catheter is visible. Thus we were able to know if the tip of a microdialysis catheter had been placed in contrast enhancing brain tissue (indicating the presence of disrupted blood–brain barrier) or non-enhancing brain tissue (indicating the blood–brain barrier was intact in that area) **[**Fig. 1**]**.

### Study design

During the surgery, after resection of tumor the neurosurgeon inserted two 70 Brain MD Catheters in residual tumor and/or peritumoral brain tissue, as was technically feasible. After a post-operative non-contrast CT scan of the brain confirmed proper placement of the intracerebral microdialysis catheters in brain tissue, the inlet tubings of the catheters were connected to portable syringe pumps (107 Microdialysis Pump, ref. no. P000127, M Dialysis) that perfused the catheters with artificial CSF at a rate of 1 µL/min.

At least 24 h after surgery, participants were given a single 1.4 mg/m^2^ dose of eribulin intravenously. To determine the neuropharmacokinetics of eribulin, dialysate samples were continuously collected from the outlet tubing of the catheters for 72 h. The microvial at the end of the catheter’s outlet tubing was changed to a new one every 60 min during the first 24 h and then every 3 h until the end of the 72 h collection period. Blood samples for assessing the relationship between systemic drug exposure and intracerebral concentrations of eribulin were obtained prior to administering the dose of eribulin, at 5, 15, 30, and 60 min, and 2, 4, 6, 8, 12, 24, 48, and 72 h, and then 1 week later. At the end of the collection period, the microdialysis catheters were removed at the bedside. Concentrations of eribulin in dialysate and plasma samples were measured by liquid chromatography/ tandem mass spectrometry.

The clinical protocol for this non-therapeutic study was approved by the City of Hope Institutional Review Board and conducted in compliance with the ethical principles of the Declaration of Helsinki, the principles of Good Clinical Practice, and all applicable regulations.

## Statistical assessments

Pharmacokinetic data were summarized by using descriptive statistics and graphical methods. The primary pharmacokinetic parameters of interest were the maximum concentration (C_max_) and area under the curve (AUC) of eribulin, measured in the dialysate and plasma samples. Differences in tumor tissue type (enhancing and non-enhancing) were assessed using linear mixed effects models, setting the intercept to be random and tissue type to be fixed, which allowed us to recognize between and within participant variability. Values from brain extracellular fluid that were below the limit of detection were set to 0.02 ng/ml.

## Results

### Patient demographics and safety

From September 2015 until February 2017, eight patients with brain tumors participated in this non-therapeutic clinical trial. Their ages ranged from 46 to 73, and the majority (5/8) were female. Five participants had brain metastases (three from non-small cell lung cancer; two from breast cancer), and three had glioblastoma (Supplementary Table 1). No unexpected toxicities from eribulin occurred. All participants tolerated well placement of the intracerebral microdialysis catheters and collection of the dialysate samples. There were no catheter-related infections or bleeding.

### Concentrations of eribulin in human brain and plasma

A total of fifteen microdialysis catheters were placed in the eight study patients. Because the majority of study participants underwent gross total resection of tumor, most of the microdialysis catheters were inserted into non-enhancing brain tissue (Supplementary Table 1). Microdialysis catheters were placed in residual enhancing tumor tissue in two of the three study participants who had glioblastoma. Only one catheter was placed in Participant 1, per the decision of the neurosurgeon. Participant 2’s post-operative imaging showed that the tip of one of the microdialysis catheters had migrated into the cerebral ventricle due to shift in brain tissue from post-operative edema. Therefore, microdialysis data from the intraventricular catheter were not included in the analysis of eribulin brain concentrations but are discussed separately below. Additionally, a post-operative brain MRI was inadvertently not done for Participant 8, and because it was not possible to determine if the catheter tips had been placed in enhancing or non-enhancing brain tissue, microdialysis data from Participant 8 were omitted from our analysis.

Eribulin plasma pharmacokinetic data from all participants are summarized in the concentration-versus-time curve in Supplementary Fig. 1. The median (minimum, maximum) eribulin plasma Cmax, AUC (0–72 h), and clearance were 466 (168, 888) ng/m, 374 (257, 1088) hr•ng/ml, and 3.16 (1.1, 5.0) L/hr/m2, respectively. The eribulin plasma pharmacokinetics in our study participants are similar to previously published data [18–20].

**Fig. 2:**
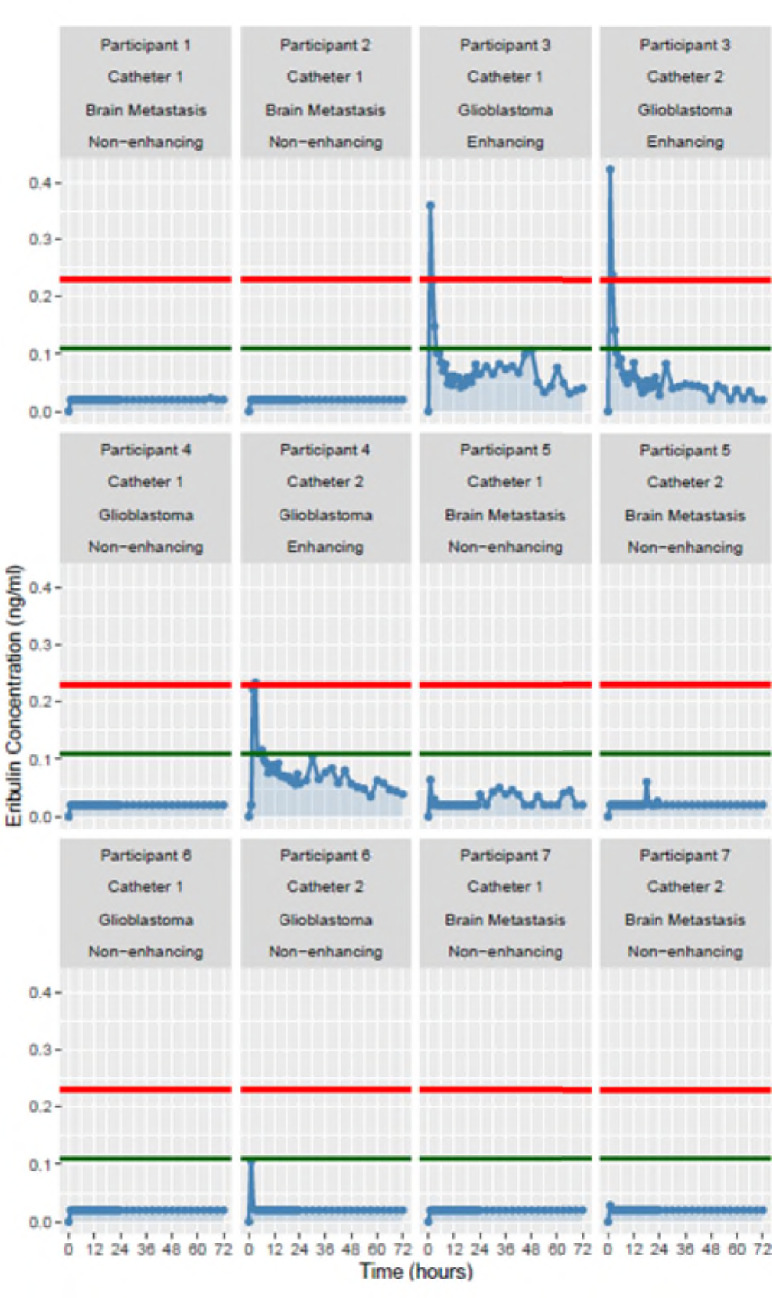
Summary of erbulin brain concentrations in study participants obtained by intracerebral microdialysis. “Non-enhancing” and “enhancing” refer to the radiographic appearance on T1 post-contrast MRI of brain tissue type (area of intact versus disrupted blood–brain barrier, respectively) in which the catheter tip was placed. The colored horizontal lines on each panel represent previously reported in vitro IC_50_ values for eribulin against two different human glioblastoma cell lines (U251; green and U87; red). [5]

The AUC of eribulin in brain extracellular fluid ranged from ≤ 1.43 to 5.12 ng/ml x hr, while plasma AUC (0–72 h) varied from 257 to 1088 ng/ml x hr. Brain to plasma ratios of eribulin ranged from 0.13 to 1.99%, The difference in mean maximum concentrations on a log_2_ scale was 3.37 (standard error = 0.59, p value = 0.005) with a significantly higher concentration of eribulin detected in enhancing tumor tissue compared to non-enhancing tissue, where the blood–brain barrier was intact **(**Fig. 2**)**.

### Concentrations of eribulin in cerebrospinal fluid

As mentioned above, dialysate samples were collected from one microdialysis catheter where the tip had migrated into the cerebral ventricle. The participant’s other microdialysis catheter was placed in non-enhancing peritumoral tissue. The AUC of eribulin in cerebrospinal fluid was 2.47 ng/ml x hr, which was higher than the AUC of eribulin in normal brain of ≤ 1.43 ng/ml x hr in this participant. In addition, the cerebrospinal fluid-to-plasma AUC ratio was 0.96%. To our knowledge, this is the first report of eribulin concentrations in cerebrospinal fluid. Although these data are from only one participant, they are consistent with what is known about differences in drug permeability between the blood-cerebrospinal fluid barrier and the blood–brain barrier [21] in that concentrations of a drug in the cerebrospinal fluid do not necessarily reflect the drug’s concentration in normal brain or brain tumor. In comparison to the overall study data, the cerebrospinal fluid concentrations of eribulin that we measured in this participant were higher than eribulin concentrations detected in peritumoral brain tissue where the blood–brain barrier was intact (AUC range: ≤ 1.43—2.06 ng/ml x hr), but lower than concentrations of eribulin measured in residual tumor tissue where the blood–brain barrier was disrupted (AUC range: 3.82–5.12 ng/ml x hr).

## Discussion

Results from our intracerebral microdialysis study indicate that eribulin does not sufficiently penetrate human blood–brain barrier, even in regions where blood–brain barrier is disrupted. As would be predicted, we found that eribulin concentrations were higher in contrast-enhancing tumor tissue where the blood–brain barrier is leaky compared to non-enhancing brain tissue where the blood–brain barrier is intact. Although the drug levels measured in enhancing brain tissue approached the reported in vitro IC_50_ for at least some human glioblastoma cells lines [5], intracerebral concentrations of eribulin were overall quite low.

It is then puzzling why the small retrospective and prospective observational studies [14–16] of treating patients with breast cancer brain metastases with eribulin documented intracranial objective response rates as high as 16%. Possibly some of these responses were actually resolution of radiation necrosis rather than true shrinkage of tumor by eribulin. Perhaps other apparent decreases in size of brain metastases were due to some participants requiring higher doses of dexamethasone to control cerebral edema between brain MRIs. Dexamethasone repairs disrupted blood–brain barrier, and so increasing the dose of dexamethasone will result in less contrast crossing into the brain, and thus enhancing brain tumors can appear smaller on an MRI if the patient is taking a higher dose of dexamethasone than when the prior brain MRI was done. Only one of the prospective studies [15] used Response Assessment in Neuro-Oncology criteria [22], which takes into account changes in dose of dexamethasone when assessing response. The other two observational studies did not provide any information about the doses of dexamethasone that participants were taking during the study period.

Limitations to our intracerebral microdialysis study include its small sample size; however, we have previously documented that only 6–10 participants are needed to characterize the neuropharmacokinetics of a drug [23, 24]. Additionally, the small number of microdialysis catheters placed in enhancing brain tissue may have hindered our ability to fully understand the extent of variability in intracerebral concentrations of eribulin in areas of brain where blood–brain barrier is disrupted. Nonetheless, one could argue that for controlling or, more importantly, preventing development of brain metastases, an understanding of the ability of a drug to cross intact blood–brain barrier is more important, and the microdialysis data from all 9 catheters placed in non-enhancing brain tissue consistently showed that sub-therapeutic concentrations of eribulin enter the brain. Another limitation to this study is that we do not have efficacy data, or rather lack of efficacy data, to correlate with our microdialysis results. Finally, because the structural integrity of blood-tumor barrier can vary between types of tumors [25] and we only measured eribulin concentrations in residual tumor of glioblastoma participants, our study results may underestimate concentrations of eribulin in brain metastases.

Our findings also highlight the inadequacy of relying on data from other species to predict the ability of a drug to cross human blood–brain barrier and achieve therapeutic concentrations in brain or brain tumor. Regardless of whether these data come from an efficacy study in mice [5] or a preclinical microdialysis study in rats using the human equivalent dose of eribulin [7], caution must be exercised when extrapolating data about blood–brain barrier and blood-tumor barrier penetrance from animal models to humans, because interspecies differences exist, particularly in regard to levels of expression and substrate specificity of various efflux transporters, such as P-glycoprotein, within the blood–brain barrier [26].

The low intracerebral concentrations of eribulin detected by the microdialysis catheters indicate that eribulin likely has minimal efficacy in treating brain metastases. The results of this study also highlight the importance of incorporating intracerebral microdialysis into early phase drug development for brain tumors. Such studies can be performed quickly, with relatively few participants, and within the context of standard surgical management of brain tumors.

## Supplementary Information

Below is the link to the electronic supplementary material.

Supplementary file1 (PDF 84 KB) Fig. S1: Eribulin plasma concentration versus time curve (n = 8). Symbols represent the mean eribulinplasma concentrations at each nominal time point and the error bars are the standard deviations

Supplementary file2 (PDF 53 KB) Fig. S2: Concentrations of eribulin in cerebrospinal fluid and non-enhancing brain tissue in which the bloodbrainbarrier was intact. These microdialysis data are from Participant 2 where one microdialysis catheter tip had migrated intothe lateral ventricle. The colored horizontal lines represent previously reported in vitro IC50 values for eribulin against twodifferent human glioblastoma cell lines (U251; green and U87; red) [5]

Supplementary file3 (PDF 23 KB)

## Data Availability

The content of this manuscript has not been published or submitted for publication elsewhere and is not under consideration elsewhere. All data generated or analyzed during this study are included in this article.
