# Supplementary figures and images for: An intracerebral microdialysis study to determine the neuropharmacokinetics of eribulin in patients with metastatic or primary brain tumors
Source: Cancer Chemother Pharmacol. 2024 Oct 18;94(6):807–13. doi: 10.1007/s00280-024-04711-2 (PMC11573798; doi:10.1007/s00280-024-04711-2)

Supplementary Figure 1.

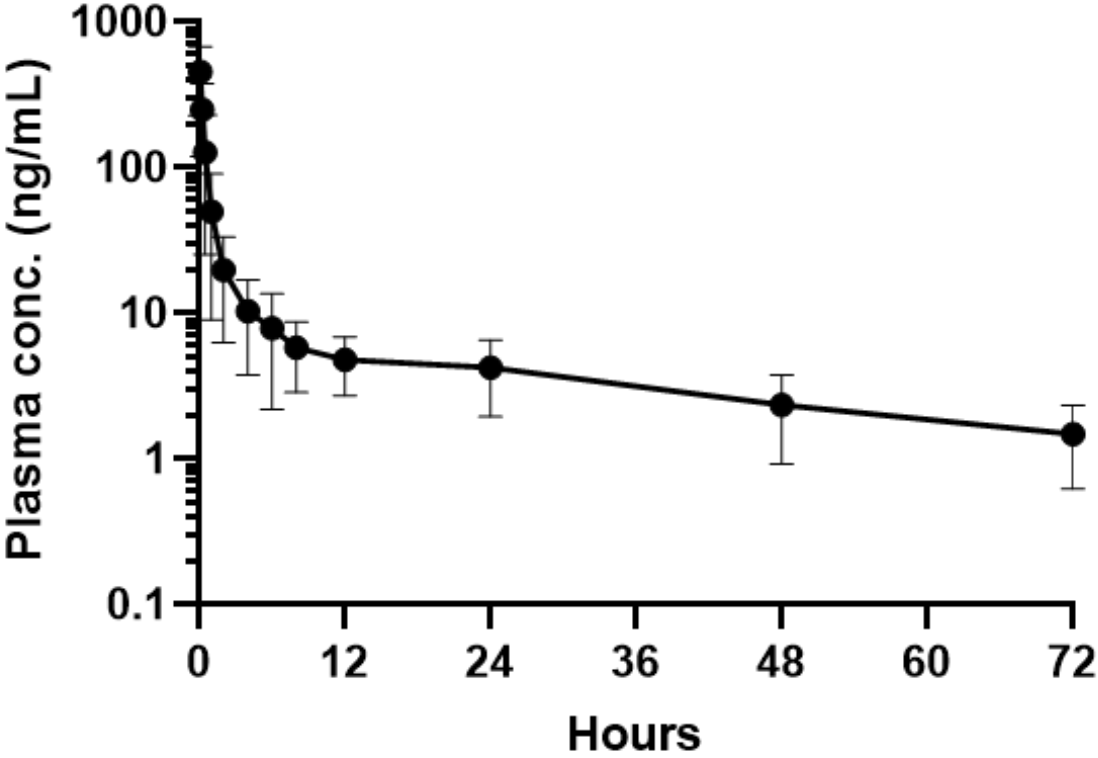

Supplement: Supplementary file 1 — Supplementary file1 (PDF 84 KB) Fig. S1: Eribulin plasma concentration versus time curve (n = 8). Symbols represent the mean eribulinplasma concentrations at each nominal time point and the error bars are the standard deviations [file 280_2024_4711_MOESM1_ESM.pdf]

Supplementary Figure 2.

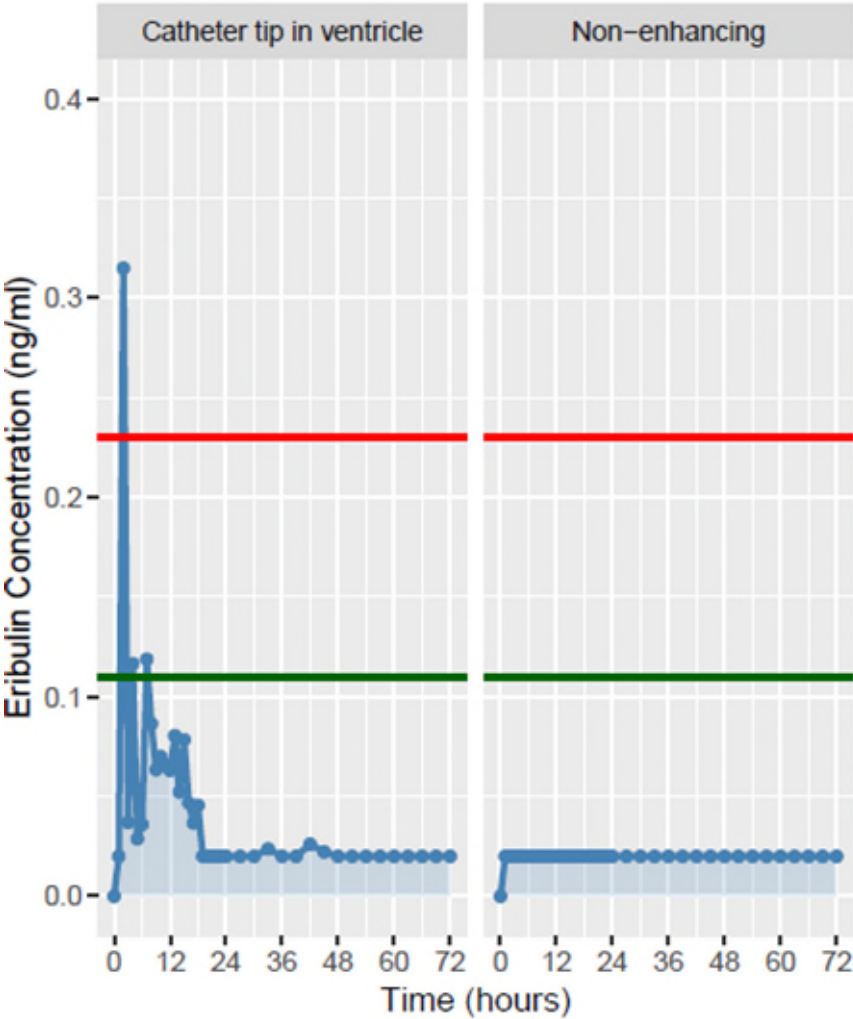

Supplement: Supplementary file 2 — Supplementary file2 (PDF 53 KB) Fig. S2: Concentrations of eribulin in cerebrospinal fluid and non-enhancing brain tissue in which the bloodbrainbarrier was intact. These microdialysis data are from Participant 2 where one microdialysis catheter tip had migrated intothe lateral ventricle. The colored horizontal lines represent previously reported in vitro IC50 values for eribulin against twodifferent human glioblastoma cell lines (U251; green and U87; red) [5] [file 280_2024_4711_MOESM2_ESM.pdf]
