# Supplementary material for: An intracerebral microdialysis study to determine the neuropharmacokinetics of eribulin in patients with metastatic or primary brain tumors
Source: Cancer Chemother Pharmacol. 2024 Oct 18;94(6):807–13. doi: 10.1007/s00280-024-04711-2 (PMC11573798; doi:10.1007/s00280-024-04711-2)
Supplement: Supplementary file 3 — Supplementary file3 (PDF 23 KB) [file 280_2024_4711_MOESM3_ESM.pdf]

Supplementary Table 1. Participant demographics

Participant number	Age	Sex	Diagnosis	Catheter tip location: type of brain tissue
1	54	FEMALE	BRC	#1 Non-enhancing
2	73	FEMALE	NSCLC	#1 Non-enhancing<br>#2 Ventricle*
3	46	FEMALE	GBM	#1 Enhancing<br>#2 Enhancing
4	54	FEMALE	GBM	#1 Non-enhancing<br>#2 Enhancing
5	56	MALE	NSCLC	#1 Non-enhancing<br>#2 Non-enhancing
6	62	MALE	GBM	#1 Non-enhancing<br>#2 Non-enhancing
7	65	MALE	NSCLC	#1 Non-enhancing<br>#2 Non-enhancing
8	48	FEMALE	BRC	Unable to verify**

Abbreviations: BRC: breast cancer. NSCLC: non-small cell lung cancer. GBM: glioblastoma

* Post-operative imaging showed the catheter had migrated into the cerebral ventricle; therefore, microdialysis data from the intraventricular catheter were not included in the analysis of eribulin brain concentrations.

** A post-operative brain MRI was inadvertently not performed; therefore, microdialysis data from this participant were not included in the analysis of eribulin brain concentrations.
